# Is handwriting constrained by phonology? Evidence from Stroop tasks with written responses and Chinese characters

**DOI:** 10.3389/fpsyg.2013.00765

**Published:** 2013-10-17

**Authors:** Markus F. Damian, Qingqing Qu

**Affiliations:** ^1^School of Experimental Psychology, University of BristolBristol, UK; ^2^Key Laboratory of Behavioral Science, Institute of Psychology, Chinese Academy of SciencesBeijing, China

**Keywords:** orthography, handwriting, lexical access, Stroop effect, Chinese

## Abstract

To what extent is handwritten word production based on phonological codes? A few studies conducted in Western languages have recently provided evidence showing that phonology contributes to the retrieval of graphemic properties in written output tasks. Less is known about how orthographic production works in languages with non-alphabetic scripts such as written Chinese. We report a Stroop study in which Chinese participants wrote the color of characters on a digital graphic tablet; characters were either neutral, or homophonic to the target (congruent), or homophonic to an alternative (incongruent). Facilitation was found from congruent homophonic distractors, but only when the homophone shared the same tone with the target. This finding suggests a contribution of phonology to written word production. A second experiment served as a control experiment to exclude the possibility that the effect in Experiment 1 had an exclusively semantic locus. Overall, the findings offer new insight into the relative contribution of phonology to handwriting, particularly in non-Western languages.

## Introduction

How is the spelling of words mentally represented? Over the last few decades, this issue has attracted substantial attention in psycholinguistic research. Much of this work has been devoted to investigations of how reading (i.e., orthographic input processing) works; relatively much less work has looked at orthographic output tasks such as spelling, writing, and typing. Furthermore, much of this research has targeted Western languages with alphabetic scripts, and little research exists which explores non-alphabetic orthographic systems such as written Chinese.

In research on orthographic word production, a central issue concerns the relative contribution of phonological codes. When individuals produce written output, is the sound of the target words involved in selecting the orthographic output codes? Early theorists (e.g., Geschwind, 1969; Luria, 1970) advocated a *phonological mediation* view according to which access to orthography is possible only via prior retrieval of sound-based codes. In other words, when an individual produces orthographic output, she first translates meaning into inner speech, and then this phonological code is transformed into orthographic representations. This view is in line with the observation that spoken language precedes written production ontogenetically and phylogenetically (e.g., Scinto, 1986). Furthermore, it fits most individuals' introspection about how writing is achieved (Hotopf, 1980), and it accounts for spelling and typing errors such as homophone substitutions (e.g., *there* spelled as “their”) and production of phonologically plausible non-words (e.g., *dearth* spelled as “dirth”; Aitchison and Todd, 1982).

However, the phonological mediation view is no longer tenable, as neuropsychological studies have demonstrated clear dissociations between spoken and written production. For instance, Bub and Kertesz (1982) reported a patient with acquired brain damage who was unable to name pictures due to a deficit at the level of the phonological lexicon (as shown by good articulation but chance-level performance in rhyme judgments on picture names and printed words), yet was able to write down picture names. Miceli et al. (1997) reported a patient who, when asked to name pictures in spoken and written form, produced consistent responses within each modality yet sometimes produced different spoken and written responses for the same picture. These and other studies suggest that, contrary to the phonological mediation view, individuals are able to access orthographic and phonological lexicons independently, rather than (as the phonological mediation position stipulates) basing access to orthography onto phonological codes.

According to the *orthographic autonomy* view (Rapp et al., 1997), individuals can access orthographic codes directly from meaning. This does not, however, exclude the possibility that phonological codes contribute to the access and retrieval of orthographic codes. Consequently, a few studies on unimpaired individuals have attempted to gather evidence regarding the relative contribution to phonology in written word production. This line of research is aided by the recent availability of inexpensive digital graphic tablets, which allow the measurement of writing latencies and other characteristics, and hence make it possible to adapt tasks and approaches from the literature on spoken word production. For instance, Bonin et al. (2001) investigated potential effects of consistency between phonological and orthographical mappings on written picture naming. Word-initial inconsistencies defined at the sublexical level affected writing latencies, with slower naming times for words with inconsistent than for those with consistent, phono-orthographic mapping. However, no difference was found when consistency was manipulated at the lexical level (heterographic homophones vs. non-homophones). This finding suggests that phonology affects orthographic production mainly via sublexical transcoding (but see Bonin et al., 1998, for a null finding concerning the role of phonology). Afonso and Álvarez (2011) adapted the popular “implicit priming“ task from the spoken literature, and observed that segmental overlap between responses words within an experimental block generated a priming effect on written latencies of Spanish participants. Crucially, this effect was eliminated when response words were orthographically related but phonologically unrelated, whereas it was preserved when response words were phonologically related but orthographically unrelated, which indicates an involvement of phonology in written production. It should be noted, however, that Shen et al. (2013) found exactly the opposite pattern in similar experiments using English participants, i.e., implicit priming was exclusively constrained by graphemic overlap, with no role of phonological overlap.

Damian et al. (2011) investigated the role of phonology in written responses using cross-modal repetition priming effects between spoken and written word production. In the literature on spoken word production, it is well documented that participants' picture naming responses are facilitated when a picture is repeatedly named, relative to the initial presentation (e.g., Cave, 1997). Theoretically, the repetition priming effect could arise from any repeated processing levels involved in spoken production, such as visual processing, conceptual activation, phonological encoding and articulation. Monsell and colleagues identified a locus of repetition priming in speaking which is independent of visual, conceptual, or phonological overlap (see Monsell et al., 1992; Wheeldon and Monsell, 1992, for details). They therefore proposed that the repetition effect in spoken production arises from a strengthening of the connection between conceptual and phonological codes. Based on this assumption, Damian et al. reasoned that if phonology is involved in written production, strengthening of the link between conceptual and phonological codes created by the prior spoken responses should facilitate subsequent written responses, hence cross modal repetition effect should appear. The authors first demonstrated parallel repetition priming effects in written and spoken responses. Additionally, they obtained cross-modal repetition priming from spoken to written production and vice versa, which implies that phonological encoding constrains preparation of written responses.

A widely used task in the literature on spoken word production is the picture-word interference (PWI) paradigm, in which participants name objects while attempting to ignore superimposed distractor words. It is well-established that form-related distractors (e.g., picture name: “bear”; distractor: “bed”) accelerate spoken picture naming relative to unrelated distractors (e.g., Rayner and Posnansky, 1978; Schriefers et al., 1990; Starreveld, 2000). A similar facilitation effect is also found when responses are written rather than spoken (e.g., Bonin and Fayol, 2000), which allows to use the task to investigate the contribution of phonology to handwritten word production. A further variable commonly manipulated in PWI tasks is the stimulus onset asynchrony (SOA) between the presentation of the picture and of the distractor. Manipulating the onset of the distractor relative to that of the picture allows researchers to tap into successive processing stages as a response is being prepared (e.g., Schriefers et al., 1990). Using this technique, Zhang and Damian (2010) showed significant priming with English participants in written latencies when distractors and picture names were orthographically as well as phonologically related (e.g., picture name: “hand”; distractor: “sand”); however, priming was small and no longer significant when distractors and picture names were closely related orthographically but less closely related phonologically (e.g., picture name: “hand”; distractor: “wand”). Degree of phonological overlap affected the size of the priming effect only at an SOA of 0 ms, whereas at an SOA of +100, priming was independent of phonological overlap and hence suggests a graphemic origin. These findings imply that phonological codes constrain access to orthographic codes, but the time-course findings additionally show that phonology might contribute to orthographic access only at a relatively early point.

Overall, the presently available evidence suggests to us that handwritten word production is indeed influenced by phonological codes (notwithstanding Bonin et al.'s, 1998, and Shen et al.'s, 2013, null findings). Indeed, a close interrelatedness between processing of spelling and sound is a plausible assumption for languages with alphabetic scripts. In non-alphabetic script systems such as written Chinese, however, it is much less obvious why orthographic retrieval should be influenced by phonological characteristics. For example, the majority (85%) of Chinese characters are phonograms (Zhu, 1988), consisting of a semantic component which provides information about a character's meaning (e.g., the character 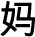, meaning “mom,” is written with the radical 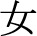, “female”) and a phonetic component which provides cues to the character's pronunciation (e.g., 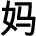, /ma1/, has the same syllable as its phonetic component 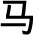, /ma3/, which means “horse”; Li and Kang, 1993). The pronunciation of a character is probabilistically related to its phonetic component, but sometimes pronunciation is entirely arbitrary; consequently, the phonetic component provides a valid pronunciation in only 38% of the characters in which they appear (Zhou, 1978). Moreover, in contrast to alphabetic systems in which graphemes map onto corresponding phonemes, phonetic radicals in Chinese characters do not correspond to specific segments of a character's phonological form. Hence, the relation between spelling and sound is to a large extent opaque, and consequently it is possible that reading of Chinese characters may be entirely unaffected by phonological properties. Contrary to this prediction, however, a growing number of studies on Chinese reading support the assumption that even in non-alphabetic languages, orthographic symbols are rapidly converted into sound-based codes (e.g., Spinks et al., 2000; Ziegler et al., 2000; see Tan and Perfetti, 1998, for a review).

The fact that spelling and sound are largely unrelated in Chinese opens the possibility of conducting experiments on handwriting with Chinese participants in order to get a better grasp on the relative contribution of phonological codes. However, to date only a single relevant study exists that we are aware of. Qu et al. (2011) recently reported the results of a PWI study in which disyllabic target picture names were written. Written distractor words were superimposed which were either phonologically and orthographically related to the picture name (i.e., shared the initial syllable and a radical on a non-initial position with the picture name, e.g., picture name: “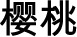,” /ying1tao2/, “cherry”; distractor: “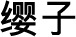,” /ying1zi/, “tassel”), were only phonologically related (i.e., shared the initial syllable but no radicals with the picture name; e.g., distractor: “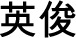, /ying1jun4/, meaning “handsome”), or were unrelated. Comparing the amount of facilitation generated by the first (phonologically and orthographically related) to the second (only phonologically related) condition indicates whether phonology contributes to handwriting of Chinese words. Priming was found for both types of distractors: phonologically and orthographically based priming was found under SOAs of 0 and +100 ms, whereas exclusively phonologically based priming was found only under an SOA of 0 ms. In other words, facilitation based on exclusively phonological relatedness was shown, but it was restricted to a relatively “early” point in time; at a later point of response preparation, priming was largely orthographically based. Note that this inference to some extent converges with the findings from Zhang and Damian (2010) with English individuals.

In the first experiment reported below, we aimed to seek converging evidence for the involvement of phonology in written production of Chinese characters. Rather than the PWI technique featured in earlier work, we used a different experimental task, adapted from the “Stroop color-word paradigm” (Stroop, 1935). In classic Stroop tasks, naming the color of incongruent color words (e.g., RED printed in blue) is slower than naming the color of control stimuli (e.g., neutral control words or solid-color squares), which is referred to as “Stroop interference”. By contrast, naming the color of congruent color words (e.g., RED printed in red) is typically faster than naming of control stimuli, referred to as “Stroop facilitation”. Furthermore, an asymmetry emerges such that when task instructions are reversed and individuals name the word rather than the color, no Stroop effect arises. The Stroop phenomenon is generally assumed to reflect the automatic lexical processing of words: incongruent color words are processed automatically and subsequently conflict with the less automatic color naming task, thus generating interference (see MacLeod, 1991, for an extensive review).

The Stroop task has been adapted to investigate the role of phonology in access to meaning from printed stimuli. In such studies using alphabetic languages, pseudohomophones of color words (e.g., “bloo”, a pseudohomophone of “blue”) are presented in a congruent ink color (blue) or an incongruent ink color (red). Previous studies have found slower color naming latencies for incongruent pseudohomophones relative to neutral controls (e.g., Dennis and Newstead, 1981), suggesting that the phonology of the pseudohomophone (“bloo”) was automatically accessed and its homophonic color word was co-activated via shared phonology, which created a conflict with the target color response. Spinks et al. (2000) used this paradigm to investigate whether the activation of phonology in access to meaning is independent of the specific nature of the orthographic system. Chinese was selected as the target language in which there are a great number of homophones and intriguingly a large number of homophones are orthographically unrelated to each other, which allows dissociating phonological effect from orthographic properties. As was the case in studies conducted in alphabetic languages, Spinks et al. observed that congruent homophones of to-be-named color word facilitated color naming whereas incongruent homophones interfered with color naming, which suggests that phonological codes are automatically activated when meaning is accessed from Chinese characters.

The classic Stroop effect is found not only with verbal, but also with manual responses (i.e., classify the target color via a response key press), and this has informed a lively debate concerning the locus of the Stroop conflict. If the Stroop effect resides at a locus prior to “response selection,” then it should be unaffected by the response format. A number of studies have reported Stroop effects with manual responses which are reduced to those found with verbal responses (e.g., Redding and Gerjets, 1977; Logan et al., 1984), although others found effects of similar size (e.g., Roe et al., 1980). The size of manual Stroop effects also appears to be influenced by whether response keys are labeled with color words, or with color patches (e.g., Sugg and McDonald, 1994), which supports the claim that verbal mediation of responses plays a role. Stroop effects have also been occasionally reported with tasks requiring an orthographic response. For instance, Logan and Zbrodoff (1998) compared verbal with typed responses (skilled typists typed the target color term of the display on a computer keyboard, and time to first keystroke was measured as latency), and reported comparable Stroop effects, with the difference between congruent and incongruent responses even larger in typed (214 ms) than in spoken (155 ms) responses. To the best of our knowledge, no versions of the Stroop task involving handwritten (rather than typed) responses has been reported in the literature, but there is no reason to surmise that such tasks would not render the typical profiles of Stroop interference and facilitation.

In the present experiment, we adapted a Stroop task to study the role of phonology in written production. In the task, participants were asked to write down the ink color of Chinese characters on a digital graphic tablet, and writing onset latencies were measured. Importantly, characters themselves never denoted color terms. Phonological overlap was manipulated such that characters were either homophonic with the target color name sharing the same tone (“congruent-same tone”), homophonic with the target color name but with a different tone (“congruent-different tone”), homophonic with a color name other than the target one sharing the same tone (“incongruent-same tone”), homophonic with a color name other than the target with a different tone (“incongruent-different tone”), or unrelated. This manipulation allowed us to explore whether written production is constrained by phonological properties, as well as to investigate the role of tonal information in Chinese. We expected to find Stroop-like interference effects forincongruent homophones, which would indicate that an incongruent color word is activated via shared homophones and created a conflict with identification of the correct color response, as was argued to be the case by Spinks et al. (2000) for spoken responses, and which is likely to be independent of response modality. The central issue we were most interested in was whether congruent homophones would facilitate written latencies on color naming, relative to the neutral baseline. If found, such Stroop-like facilitation effects would provide the strongest evidence to date that phonology is activated when orthographic representations are accessed in written production: as the distractor is presented visually, the response involves handwriting, and graphemic overlap between character and written response is entirely avoided, priming due to phonological overlap (in this case, homophony) would support the claim that phonology is involved in the generation of written words.

Moreover, we varied the SOA between the presentation of the color and the character. Manipulating the onset of distractor (the character in this case) relative to that of the target could provide insight into the time course of an effect, and in the Stroop literature a rich literature on the effects of SOA manipulations exists (for instance, see Glaser and Glaser, 1982, for seminal work, and MacLeod, 1991, for an extensive review). As summarized above, previous findings (Zhang and Damian, 2010; Qu et al., 2011) had suggested that phonological activation in writing takes place at relatively early stages. Therefore, in the present experiment, in addition to an SOA of 0 ms, two further “negative” SOAs (−300, −150 ms) were included, hypothesized to tap into earlier processing stages of response generation. Under these SOAs, the character was first presented in black color, and then changed to the target color after the appropriate time interval (300 or 150 ms). However, inclusion of a range of SOAs was mainly done in order to avoid making Type II errors concerning an effect of phonology (i.e., failing to include an SOA under which an effect might have been found), and we had no strong predictions concerning the time course of a hypothetical effect. Hence, under any SOA, if the congruent homophones facilitate writing latencies of color naming, this would provide further evidence for the involvement of phonology in written production.

## Experiment 1

### Methods

#### Participants

Thirty-seven native Mandarin Chinese speakers, recruited from the student population at the University of Bristol, participated in the experiment. All were writers of simplified Chinese characters. They were paid a small fee for participation. All had normal or corrected-to-normal vision and no history of dysgraphia.

#### Materials and design

Four colors (purple, green, red, and blue) were used as target responses to be written. All four color words were monosyllabic in Chinese and hence were written as a single character (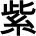, /zi3/, purple; 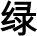, /lü4/, green; 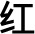, /hong2/, red; 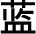, /lan2/, blue). Twelve monosyllabic Chinese characters were selected which were homophonic to color words while sharing the same tone, with three characters for each color (“congruent-same tone”, “
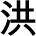
”, /hong2/, flood,—“
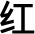
”, /hong2/, red). A further 12 mono-syllabic Chinese characters homophonic to the color words but with a different tone were selected, again, three characters for each color (“congruent-different tone”, “
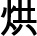
”, /hong1/, drying,—“
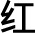
”, /hong2/, red). In the two congruent conditions, any orthographic or semantic relation between color words and homophones was avoided. To form the respective incongruent conditions, the homophonic characters in each congruent condition were then recombined with the color words so that each character was homophonic with a different color name sharing the same tone (“incongruent-same tone”, “
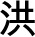
”, /hong2/, flood—“
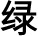
”, /lü4/, green) or with a different tone (“incongruent-different tone”, “
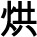
”, /hong1/, drying—“
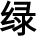
”, /lü4/, green). In the incongruent conditions, any phonological, orthographic or semantic overlap between the color words and incongruent homophones was avoided. In this way, 12 trials for each of the four conditions were formed, hence generating 48 critical trials.

Additionally, 24 neutral characters which were orthographically and phonologically unrelated to all four color words were selected as the baseline condition. By including trials with neutral characters, the percentage of critical trials was decreased which could reduce the possibility that participants develop strategies to respond. As was the case for the critical trials, each of 24 neutral characters was paired with two colors, thus producing 48 neutral trials. In order to directly assess effects arising from each of two types of homophones, same-tone homophones, different-tone homophones and neutral characters were matched on the number of strokes and character frequency. The lexical properties of same-tone homophones, different-tone homophones and neutral characters are shown in Table 1. A complete list of experimental materials is presented in Appendix.

**Table 1:** **Lexical properties of distractor characters used in Experiment 1 and 2**.

	**Same-tone homophones**	**Different-tone homophones**	**Neutral control**
Character frequency (log)^*^	3.8	3.8	3.8
Stroke number	8.8	8.8	8.6

* Taken from Chinese Corpora, www.cncorpus.org.

Character-color SOA was manipulated as −300, −150, and 0 ms. At SOA = −300 and −150 ms, a character was first presented in black for 300/150 ms, and then immediately replaced by the same character in color. At SOA = 0 ms, a colored character was presented straightaway. Under each SOA, all 24 congruent trials, 24 incongruent trials and 48 neutral trials were presented, thus forming 96 trials in each SOA (288 trials in total). Trials were blocked by SOA; and the order of SOA blocks for each participant was determined by a Latin square design. A new pseudorandom trial order was generated for each block and participant, with the constraint that neither colors nor characters were repeated on consecutive trials.

#### Apparatus

The experiment was run using DMDX (Forster and Forster, 2003) from an IBM-compatible computer on a 17-in. monitor. Response latencies, i.e., the interval between onset of the color dimension and initial contact of the pen with the tablet, were recorded by a WACOM Intuos A4 graphic tablet and a WACOM inking pen. Participants wrote down their responses on an A4 sheet of paper attached to the tablet. A sheet of paper consisted of 96 lines (4 columns × 24 lines), which corresponds to one SOA block. Characters presented in 36 point Song font were displayed at the bottom of the screen to reduce participants' head and eye movements as they wrote responses.

#### Procedure

Participants were first instructed to hold the pen slightly above the corresponding line to get ready for writing down responses so that initiation of the response would not require an arm movement; neither should they drop the pen on the sheet before identifying responses. Compliance with these instructions was assured before the experiment began. They were asked to write down the color of characters as quickly and accurately as possible. In a subsequent practice block, 12 neutral characters in colors (3 characters per color) were presented. Then, three SOA blocks of 96 trials each were carried out. On each trial, participants saw a sequence consisting of a fixation cross (500 ms), a blank screen (500 ms), and a character, and an inter-trial interval (1000 ms). As described above, at SOA = 0 ms, a colored character was displayed directly, whereas at SOA = −300 and −150 ms, a blank character was first presented and then, after 300/150 ms replaced with the same character in a particular color. Colored characters remained on the screen for 3000 ms and response latencies were measured during this period. Subsequently, the colored character disappeared and the next trial began. The whole experiment took approximately 30 min.

### Results

Latencies for incorrect responses (0.5%) were excluded from analysis, and latencies faster than 200 ms or slower than 2000 ms (1.4%) were discarded as outliers. Table 2 presents the mean latencies and error percentages of responses for each condition. Stroop-like facilitation and interference effect were calculated by subtracting response latencies in the congruent and incongruent conditions respectively from those in the neutral condition. Latencies and errors were analyzed separately for Stroop-like facilitation and interference, and for homophones with the same and different tone. The results were analyzed using a linear mixed effects model approach (Bates, 2005; Baayen et al., 2008) that included fixed categorical effects of Congruity (Congruent/Incongruent, Neutral), and SOA (−300, −150, 0 ms) and by-participant and by-item random intercepts. Models were fit to the data using a restricted maximum likelihood technique. Model fitting was carried out by initially specifying a model that only included the random factors (participants and items, i.e., color responses) which was then enriched by subsequently adding the fixed factor Congruity, followed by SOA, and finally the interaction between the two factors. The best fitting model was defined to be the most complex model that significantly improved the fit over the previous model.

**Table 2:** **Experiment 1: mean response latencies and error rates (PE) as a function of congruity, character type and stimulus onset asynchrony (SOA)**.

**Condition**	**SOA (in ms)**					
	**−300**		**−150**		**0**	
	**RT (PE)**	**Effect**	**RT (PE)**	**Effect**	**RT (PE)**	**Effect**
Neutral	834 (0.4)		833 (0.5)		852 (0.5)	
**CONGRUENT**						
Same tone	807 (0.0)	+27^**^ (+0.4^†^)	834 (0.9)	−1 (−0.4)	860 (0.0)	−8 (+0.5^*^)
Different tone	834 (0.2)	0 (+0.2)	823 (0.2)	+10 (+0.3)	837 (0.5)	+15 (0.0)
**INCONGRUENT**						
Same tone	846 (0.5)	−12 (−0.1)	852 (0.9)	−18^**^ (−0.4)	892 (0.1)	−40^**^ (+0.4)
Different tone	854 (0.2)	−20^*^ (+0.2)	846 (0.7)	−13^*^ (−0.2)	871 (1.4)	−19^†^ (−0.9^†^)

** p < 0.01;

* p < 0.05;

† 0.05 < p < 0.1.

#### Stroop-like facilitation effect

***Congruent-same tone*** Including Congruity did not significantly improve the fit, χ^2^_(1, *N* = 6533)_ = 0.94, *p* = 0.331^1^. The best fitting model included SOA and the interaction between Congruity and SOA, χ^2^s > 7.70, *p* ≤ 0.005. Planned comparisons that assessed the effects of congruity at each SOA separately showed a highly significant facilitation at SOA = −300 ms, *t*_(2155)_ = 3.21, *p* = 0.001, but not at SOA = −150 and 0 ms, *t*s < 1, *p*s ≥ 0.42.

***Congruent-different tone.*** The best fitting model included SOA only, χ^2^_(1, *N* = 6538)_ = 20.29, *p* < 0.001. Including Congruity and the interaction did not improve the fit, χ^2^s ≤ 1.87, *p*s ≥ 0.17. Tests that assessed the effects of congruity separately showed no significant facilitation at each SOA, *t*s ≤ 1.58, *p*s ≥ 0.114.

#### Stroop-like interference effect

***Incongruent-same tone.*** The best fitting model included Congruity, SOA and the interaction between Congruity and SOA, χ^2^s_(1, *N* = 6528)_ ≥ 4.38, *p*s ≤ 0.036. Planned comparisons that assessed the effects of congruity at each SOA separately showed highly significant interference effects at SOA = 0 ms, *t*_(2184)_ = 4.37, *p* < 0.001 and at SOA = −150 ms, *t*_(2189)_ = 2.58, *p* < 0.01; but not at SOA = −300 ms, *t*_(2153)_ = 1.32, *p* = 0.19.

***Incongruent-different tone.*** The best fitting model included Congruity and SOA, χ^2^s_(1, *N* = 6526)_ ≥ 13.80, *p*s < 0.001. Including the interaction between Congruity and SOA did not improve the fitting, χ^2^ < 1. Planned comparisons that assessed the effects of congruity at each SOA separately showed significant interference effects at SOA = −150 ms, *t*_(2187)_ = 2.55, *p* = 0.011 and at SOA = −300 ms, *t*_(2189)_ = 2.27, *p* = 0.023, and a marginally significant interference effect at SOA = 0 ms, *t*_(2183)_ = 1.89, *p* = 0.059.

A parallel analysis was conducted on the errors, but a binomial family was used because of the binary nature of the data (Jaeger, 2008). No main effects or interactions reached significance for any condition, *Wald Zs* ≤ 1.58, *p*s ≥ 0.110. Planned comparisons showed a significant facilitation effect in the “congruent-same tone” condition at SOA = 0 ms, *p* = 0.044, marginally significant facilitation at SOA = −300 ms, *p* = 0.077, and marginally significant interference in the “incongruent-different tone” condition at SOA = 0 ms, *p* = 0.09. All other comparisons were not significant, *p*s > 0.17.

### Discussion

The results showed that writing latencies on color naming were slower for incongruent trials relative to neutral control trials at all three SOAs (except for the “incongruent-same tone” condition under SOA = −300 ms), and this was the case irrespective of whether or not characters and color names shared the tone. This interference effect likely reflects the fact that in the incongruent conditions, Chinese writers activated the phonological information of the character and co-activated an incongruent color name via shared phonology, thus creating a conflict with the selection of the to-be-written color response. More interesting with regard to the aim of identifying the contribution of phonology to writing is the observation that congruent homophones of color characters produced facilitation in response latencies at SOA = −300 ms, and error rates were significantly or marginally facilitated at SOA = −300 and 0 ms. This constitutes evidence for the claim that phonological codes affect the production of written words. Moreover, this facilitation effect was restricted to homophones which shared the tone with the target color. A mere syllabic specification, in the absence of tonal overlap, was not capable of producing a parallel facilitation effect, suggesting that tone is an essential component of Chinese phonological representations. We will return to the issue of the role of tonal representations in the General Discussion.

Our account of the facilitatory effect of congruent homophones in the first experiment hinges on the idea that written target responses are partially based on phonological codes, and are therefore primed by a phonologically overlapping distractor dimension. There is, however, an alternative explanation of why facilitation effects could have emerged in Experiment 1. Arguably, the critical characters with homophonic names evoke multiple meanings, among them the corresponding color sense, and the latter could match or mismatch the color response at the conceptual level. According to this argument, a character in the “congruent-same tone” such as “
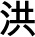
”, /hong2/, activates not only its meaning “flood” but also the meaning of its homophone /hong2/, “red”. The primary task - written naming of the color of the character—requires identification of the target color and therefore its meaning. If this meaning is already preactivated via homophony of the character (see above), a facilitation effect might emerge. If so, this effect would indicate priming at the conceptual level, but it might tell us relatively little about the role of phonology in handwriting.

Whether this scenario is plausible is *prima facie* difficult to determine, but it critically relates to an extensive literature on the locus of Stroop effects. In the Stroop domain, the locus of Stroop facilitation and (more prominently) interference has been controversially discussed for many decades (see MacLeod, 1991, for a review of competing theories). Most accounts (e.g., Morton and Chambers, 1973; LaBerge and Samuels, 1974; Posner and Snyder, 1975; Shiffrin and Schneider, 1977) stipulate that Stroop effects arise from some form of conflict at the stage of response selection, i.e., a conflict between color and word at the level of meaning is by itself not sufficient to generate the effect, but rather response selection of the target dimension is affected by the word. However, it has occasionally been proposed that the Stroop effect might be based on semantic, rather than response, competition (e.g., Seymour, 1977). In the typical Stroop manipulation, semantic and response compatibility are confounded: the word “green” printed in red is semantically related to (i.e., conflicts with) the target, but it also supports the incorrect response. Numerous attempts have been made to disentangle the two dimensions. For instance, “matching tasks” have been introduced in which rather than naming the ink of a color word, participants are presented with a color word and a color bar, and are instructed to categorize via a key press whether or not the two dimensions match (e.g., Dyer, 1973; Luo, 1999; Goldfarb and Henik, 2006; see Treisman and Fearnley, 1969, for an early version of this task which required card sorting). The reasoning is that in such a “meaning decision task,” response selection does not require selection among alternatives corresponding to competing color terms, and hence Stroop interference effects in such tasks would indicate a locus of semantic, rather than response, competition. The resulting pattern of findings is complex, and its interpretation remains controversial (see the debate between Luo, 1999 and Goldfarb and Henik, 2006, regarding the theoretical inferences that can be drawn from such tasks).

In the second experiment, we adopted an approach in which the handwritten responses of the first experiment were replaced with key press responses. We did not expect to resolve the extensive debate on the locus and origin of the Stroop effect, but rather devised a control experiment which attempted to replicate our first experiment, and critically the homophone manipulation, as closely as possible while changing response mode. As outlined in the Introduction, Stroop effects are generally found not only when participants name the target color, but also when they classify colors via key presses. For instance, Logan reported congruent-incongruent differences of 155 ms for verbal responses, of 214 ms for typed responses, and of 138 ms for responses involving key presses. The size of Stroop effects in manual response tasks varies somewhat across studies (e.g., Redding and Gerjets, 1977; Roe et al., 1980; Logan et al., 1984) but the general pattern is that effects are still substantial with key press responses. We reasoned that if the Stroop interference found in our first experiment is due to co-activation of multiple senses evoked by a homophonic distractor, then it should be independent of response mode, and so should also emerge in a control experiment involving key presses as responses. Critically, we attributed the same-tone homophonic facilitation effect obtained in our first experiment to a mechanism which primes a phonologically mediated graphemic response (and hence is informative with regard to the properties of handwriting). If so, then this effect should disappear in the second experiment because here, generation of handwritten codes, or indeed, any verbal codes, is no longer required. In our second experiment, materials were hence the same as in the first experiment, but now participants categorized the target colors with manual key presses rather than written responses.

## Experiment 2

### Methods

#### Participants

Twenty-four native Mandarin Chinese speakers from Beijing Forestry University and China Agricultural University, none of whom had been in the first experiment, participated and were paid a small fee. All were writers of simplified Chinese characters, had normal or corrected-to-normal vision and no history of dysgraphia.

#### Materials and design

The materials listed in Appendix again served as stimuli. Participants responded to target colors “red” and “green” with one key press, and to targets “blue” and “purple” with another. Same- and different tone homophone characters were combined with the targets such that they formed four congruent and incongruent conditions (12 trials for each of the critical four conditions). As in Experiment 1, each of 24 “neutral” characters was paired with two colors, creating 48 neutral trials. Additionally, each of the same- and different tone homophone characters was paired with an unrelated color to create 24 filler trials, thus forming 120 trials in each SOA (360 trials in total). As in the first experiment, three SOAs (−300, −150, and 0 ms) were used. Within each SOA block, each color-character combination was presented once in pseudo-random order, with the constraint that neither colors nor characters were repeated on consecutive trials.

#### Apparatus

Stimuli were presented using DMDX. Participants indicated their response by pressing the “L” or the “A” key on the computer keyboard. Response key assignment was rotated across participants.

#### Procedure

Participants were tested either alone or in pairs. They were instructed that on each trial they would see a colored Chinese character, and that they should attempt to ignore the character and press one response key if the color was red or green, and another response key if the color was blue or purple. In a subsequent practice block, 12 neutral combinations were presented, in which each target color was present three times. Then, three SOA blocks of 120 trials each were carried out. Breaks were provided between blocks. The whole experiment took approximately 30 min per participant.

### Results

Latencies for incorrect responses (2.5%) were excluded from analysis, and latencies faster than 200 ms or slower than 1400 ms (0.1%) were discarded as outliers. Table 3 presents the mean latencies and error percentages of responses for each condition.

**Table 3:** **Experiment 2: mean response latencies and error rates (PE) as a function of congruity, character type and stimulus onset asynchrony (SOA)**.

**Condition**	**SOA (in ms)**					
	**−300**		**−150**		**0**	
	**RT (PE)**	**Effect**	**RT (PE)**	**Effect**	**RT (PE)**	**Effect**
Neutral	501 (1.9)		512 (2.6)		492 (2.1)	
**CONGRUENT**						
Same tone	497 (2.8)	+4 (−0.9)	513 (2.8)	−1 (−0.2)	485 (3.8)	+7 (−1.7^†^)
Different tone	512 (1.7)	−11 (+0.2)	504 (2.8)	+8 (−0.2)	510 (1.7)	−18^*^ (+0.4)
**INCONGRUENT**						
Same tone	531 (3.1)	−30^**^ (−1.2)	533 (2.8)	−21^**^ (−0.2)	519 (4.5)	−27^**^ (−2.4^*^)
Different tone	522 (2.1)	−21^**^ (−0.2)	537 (3.1)	−25^**^ (−0.5)	506 (3.1)	−14^*^ (−1.0)

** p < 0.01;

* p < 0.05;

† 0.05 < p < 0.1.

Data were analyzed in accordance with the first experiment: facilitation was computed as “neutral” minus “congruent-same response,” and interference as “neutral” minus “incongruent-different response.” Both types of effects were calculated separately for the “same” and “different” tone conditions.

#### Stroop-like facilitation

***Congruent-same tone.*** The best fitting model included SOA only, χ^2^_(1, *N* = 4213)_ = 6.48, *p* = 0.011. Additionally including Congruity, or including the interaction between Congruity and SOA, did not significantly improve the fit, χ^2^s ≤ 0.43, *p* ≥ 0.511. Planned comparisons that assessed the effects of congruity under each SOA separately showed no significant results, χ^2^s ≤ 1.01, *p* ≥ 0.320.

***Congruent-different tone.*** The best fitting model included SOA only, χ^2^_(1, *N* = 4223)_ = 3.88, *p* = 0.049. Additionally including Congruity marginally improved the fit, χ^2^ = 2.75, *p* = 0.097. Including an interaction between SOA and Congruity did not further improve the fit, χ^2^ = 0.52, *p* = 0.472. Planned comparisons showed a significant effect at SOA = 0 ms, χ^2^ = 6.22, *p* = 0.013, but not under the other SOAs, χ^2^s ≤ 2.22, *p* ≥ 0.137. Note that the significant effect at SOA = 0 ms is interfering rather than facilitatory.

#### Stroop-like interference

***Incongruent-same tone.*** The best fitting model included both Congruity and SOA as main effects, χ^2^ ≥ 5.11, *p* ≤ 0.024. Including an interaction between SOA and Congruity did not further improve the fit, χ^2^ = 0.00, *p* = 0.979. Planned comparisons showed significant effects at SOA = −300 ms, χ^2^ = 13.74, *p* < 0.001, at SOA = −150 ms, χ^2^ = 7.91, *p* = 0.005, and at SOA = 0 ms, χ^2^ = 12.65, *p* < 0.001.

***Incongruent-different tone.*** The best fitting model included both Congruity and SOA as main effects, χ^2^ ≥ 6.31, *p* ≤ 0.012. Including an interaction between SOA and Congruity did not further improve the fit, χ^2^ = 0.22, *p* = 0.642. Planned comparisons showed significant effects at SOA = −300 ms, χ^2^ = 7.63, *p* = 0.006, at SOA = −150 ms, χ^2^ = 10.33, *p* = 0.001, and at SOA = 0 ms, χ^2^ = 4.84, *p* = 0.028.

A parallel analysis was conducted on the errors, using a binomial family. Regarding Stroop facilitation, no main effects or interactions reached significance, Wald *Z*s < 2.47, *p*s > 0.116. Planned comparisons showed a marginally significant effect for the “same tone” condition at SOA = 0 ms, χ^2^ = 2.81, *p* = 0.094 (note that this effect is interfering rather than facilitatory), but not for any of the other comparisons, Wald *Z*s < 0.83, *p*s > 0.363. Regarding Stroop interference, in the “same tone” condition the best fitting model included Congruity, χ^2^_(1, *N* = 4320)_ = 4.42, *p* = 0.036, but neither inclusion of SOA, nor an interaction between Congruity and SOA, further improved the model, χ^2^ ≤ 0.54, *p* ≥ 0.463. For the “different tone” condition, no main effects or interactions reached significance, Wald *Z*s < 1.00, *p*s > 0.312. Planned comparisons showed a significant effect in the “same tone” condition at SOA = 0 ms, χ^2^ = 4.94, *p* = 0.026. None of the other comparisons reached significance, Wald *Z*s < 1.51, *p*s > 0.219.

### Discussion

In this experiment, highly significant Stroop interference was found, both for homophones sharing the same tone with targets, and those without. These results are similar to those found in our first experiment, and suggest that the locus of this interference effect does not depend on response mode. Indeed, given that Spinks et al. (2000) had reported similar Stroop interference from Chinese characters with *verbal* responses, the results suggest either a locus prior to response selection, or the possibility that response selection in both written and manual responses relies on the same codes. We believe that in line with Spinks' et al. account, the most likely explanation is that a homophonic character will evoke its multiple senses, and that the color sense will conflict with an incongruent color response.

By contrast, the facilitatory effect found in the first experiment for homophones sharing the same tone with the target (but not for homophones not sharing their tone) is evidently specific to written responses (or rather, responses relying to some extent on phonological codes) because it was not found in the control experiment requiring key presses rather than written responses. Significant *interference* was found for congruent distractors at SOA = 0 ms, but only for distractor homophones which did not share their tone with the target. We have no ready explanation for this finding (other than it might be a type I error). However, from a broader perspective, the contrast between the two experiments regarding the effects of (same-tone) congruent homophones further supports our claim that the facilitatory effect shown in the first experiment reflects the involvement of phonological codes in an orthographically based output task.

## General discussion

The first experiment reported in this article investigated whether phonology constrains written word production. We used a version of the well-known Stroop task which required written rather than spoken responses, and we tested native Chinese speakers. Colored Chinese characters were presented and participants wrote the color name on a digital tablet while attempting to ignore the character itself. Compared to neutral characters which stood in no obvious relationship to the color response, for *incongruent* trialslatencies were slower at all three SOAs (except for the “incongruent-same tone” condition under SOA = −300 ms), and interference was independent of whether or not characters and color names shared the tone. However, interference depended to some extent on SOA, with “same tone” interference about twice as large as “different tone” interference at SOA = 0 ms, less of a discrepancy by tone at SOA = −150 ms, and rather the reverse pattern (numerically larger interference for the “different” than the “same” tone condition) at SOA = −300 ms. More importantly, relative to the neutral condition, characters which were *congruent* (i.e., homophonic with the target response) facilitated written color naming latencies, but only when they shared the same tone, and mainly at a large negative SOA (−300 ms).

The maximum Stroop interference obtained in our first experiment was 40 ms, and the maximum facilitation was 27 ms. As stated in the Introduction, we are not aware of Stroop tasks requiring handwritten responses described in the literature, so it is somewhat difficult to put these effect sizes into context. However, it is informative to compare these effects with the results previously reported by Spinks et al. (2000) because these authors used very similar materials to ours, but participants named the target colors in spoken form. Spinks et al. used only a single SOA (0 ms) whereas we assessed a range of SOAs; however, Spinks et al. also included directly congruent and incongruent color characters (rather than color homophones), which allows to test for the presence of standard Stroop effects. Across two experiments, Spinks et al. reported interference effects from incongruent distractors of 29 ms when they shared the tone with an (incongruent) color term, and of 6 ms when they did not share tone. They also reported facilitation effects from congruent homophone distractors of 49 ms when they shared the tone with a color and of 39 ms when tone differed. Hence, the size of our effects is generally in line with was previously reported. It must be noted that these effects are generally small, compared to “standard” Stroop effects. Spinks et al. found direct (non-homophonic) Stroop facilitation of 80 ms, and Stroop interference of 68 ms. As stated above, we are not aware of existing Stroop studies with handwritten responses, but with typed rather than handwritten responses and English participants, Logan and Zbrodoff reported Stroop interference of 176 ms, and facilitation of 38 ms. In a comparable study, Damian and Freeman (2008) found Stroop interference of 125 ms, and facilitation of 64 ms. In combination, these findings show that with Chinese characters with are homophones of color terms, Stroop effects are substantially reduced relative to “standard” Stroop effects. Of course, this is to a large extent predicted, because effects arise not via a direct conflict between target and distractor dimension, but are rather mediated via shared phonology.

The interference observed both by Spinks et al. (2000) and by us likely reflects the fact that Chinese individuals involuntarily activated the phonological information of the character and co-activated an incongruent color name via shared phonology, thus creating a conflict with the selection of the to-be-written color response. In other words, printed characters are rapidly recoded into phonological format, and multiple senses corresponding to the homophones are activated. The findings more specifically speak to the nature of phonological representations in Chinese. Dissimilar to most Indo-European languages, Mandarin Chinese is a tonal language including four separate tones. Tone is lexically distinctive; and characters sharing the same segmental information but with different tones specify different words which almost always have completely different meanings, e.g., 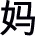, /ma1/, “mother” vs. 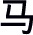, /ma3/, “horse”. It has long been controversial whether or not the specification of tone is an essential part of phonological activation (see Introduction). Based on the fact that under the single SOA (0 ms) included in Spinks et al.'s study, Stroop interference depended on whether or not a distractor homophone shared the tone with a color, the authors argued that “a Chinese character's phonological code appears to include both phonetic information (consonant and vowel) and tonal information, in effect a full phonological specification of the character” (p. B7). This inference dovetails with results reported by Xu et al. (1999) in a semantic relatedness judgment task: a cue word was presented (e.g.,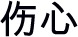, /shang1xin1/, sad) followed by a test word which was either a synonym of the cue word (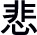, /bei1/, “sad”) or a homophone of the synonym with the same (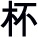, /bei1/, “cup”) or different tone (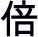, /bei4/, “times”). Participants were asked to judge whether or not the two words presented were semantically related. Results showed that participants were slower to respond with “no” when the test word was homophonic to the synonym of the cue word than when they were unrelated. Importantly, this interference effect was restricted to homophones sharing the same tone, which suggests that tonal information is an essential part of Mandarin phonological representations. However, conflicting evidence by Taft and Chen (1992) must be noted: when Chinese speakers judged whether or not two characters sounded the same, they encountered difficulties on negative responses even when the characters shared the same syllable but differed in tone.

Our own study provides additional evidence to this debate: whether or not homophonic distractor characters share the tone with a color competitor to the target is clearly relevant. However, interpretation of this pattern is rendered somewhat complex when SOAs are manipulated in a Stroop task, and here results are less clear-cut than those from the single SOA included in Spinks et al.'s study. At SOA = 0 ms, different-tone interference was indeed substantially reduced relative to same-tone interference, a finding which highlights the role of tonal information and hence converges with the one reported by Spinks et al. However, at the negative SOAs the pattern was much less clear, with similar (SOA = −150 ms) or even more (SOA = −150 ms) for different- than for same-tone incongruent distractors. The conventional interpretation of SOAs is that negative SOAs allow more time for distractor processing, hence tapping into “earlier” stages of target dimension processing (cf. MacLeod, 1991). If this is accepted, a possible inference could be that at early stages of form encoding, tone is less relevant than at later ones. However, we would caution against over interpreting the present results in this manner. At minimum, our findings underscore the importance of including multiple SOAs in Stroop studies, because results from just a single SOA might lead to incorrect or incomplete interpretations.

The central question of our study, however, concerned a potential contribution of phonology to handwritten responses. Given that the task required an orthographic response, and printed distractor and written response never shared any graphemic codes, we reasoned that a *facilitatory* effect based on homophony of a distractor with a color term would constitute evidence for the claim that written production is supported by phonological codes. As reviewed in the Introduction, a limited (but slowly growing) literature supports this claim. However, because in Western languages, orthographic and phonological codes are inevitably confounded, it is very difficult to design experiments which would clearly attribute an experimental effect to one source or another. A non-alphabetic script such as written Chinese allows a clean dissociation between orthographic and phonological codes, allowing a better investigation of the role of phonology in written tasks (or conversely, the role of orthography in spoken tasks; e.g., Bi et al., 2009). To date, this has been attempted only in a single published study (Qu et al., 2011) using a picture-word technique. If the line of argument presented above is accepted, the results of our first experiment substantially strengthen the existing evidence for a phonological influence in orthographic word production. Hence, some form of sub-semantic mapping between phonological and orthographic codes must mediate the facilitation effect. In models of reading, a sublexical route which supports a grapheme-to-phoneme conversion has long been a standard assumption (e.g., Coltheart et al., 2001), and similar conversion routes, but in the opposite direction, have been advocated in of models of spelling (e.g., Barry and Seymour, 1988) and handwriting (e.g., Bonin et al., 2001). However, a division into lexical and sublexical routes is not tenable in written Chinese, because orthography and phonology are only very indirectly related. Mappings are therefore likely to exist at relatively high representational levels, such as characters-to-syllables. Such syllable-level mappings would naturally account for our results in Experiment 1, but this does not exclude the possibility of additional mappings between other types of representations (strokes, radicals, rimes, tones, etc.; see Weekes et al., 2006). Research on this topic is only in its infancy, and more work is clearly needed to establish the nature of orthography-phonology correspondences in Chinese.

As in the case for the Stroop interference effects (see previous section), the role of tone must be considered. Stroop facilitation at SOA = −300 ms was restricted to homophonic distractors sharing a tone with the (target) color term, but it was absent in homophones with a different tone. This finding suggests, to some extent in line with the inference drawn from the Stroop interference effects, that tonal information is an essential part of Mandarin phonological representations. It is difficult to disagree with Spinks et al.'s (2000) assertion that a Chinese word's phonological code tightly integrates both phonetic and tonal information, although recently there has been a debate about the exact nature of the phonological representations of spoken Mandarin (O'Seaghdha et al., 2010, 2013; Qu et al., 2012, 2013). We are at present not aware of theoretical or computational models of Chinese word production which implement detailed assumptions about how tonal information is represented and combined with phonological codes. Again, more work on spoken and written word production is needed to compare and delineate models of Western (e.g., WEAVER; Levelt et al., 1999) and non-Western language production.

A secondary, but nevertheless important, element of the results of our first experiment concerns the time course of phonological activation in access to orthographic codes shown in Experiment 1. Qu et al. (2011) used a picture-word technique with Chinese participants and with a similar logic to the current Experiment 1, and found phonologically based priming only at “early” SOAs, whereas at “later” SOAs, priming was mainly graphemically based. On the assumption that the manipulation of the SOA in PWI studies allows insight into the picture-naming process as it unfolds over time, this result suggests that effects of phonology are restricted to relatively early processing stages of graphemic encoding. Similarly, Zhang and Damian (2010) conducted a picture-word study with English speakers, and also found a relatively “early” effect of phonology. Together, these studies might suggest that in an orthographic word production task, target concepts activate phonological and orthographic codes in parallel, but perhaps with phonological codes being accessed more rapidly than orthographic ones. If the activated sound codes are subsequently converted to orthographic representations, they may affect earlier stages of graphemic encoding. At a later point in time, the impact of the phonological route becomes less relevant, and priming is mainly orthographic. In our current Experiment 1, congruent (same tone) homophones similarly facilitated written responses only at an “early” SOA (−300 ms), but not at −150 and 0 ms. Comparing SOA curves across different tasks and languages is not straightforward. Nevertheless, a common pattern emerges such that phonological constraints on written word generation take place at relatively early processing stage. Further research, perhaps involving the measurement of electrophysiological (EEG) responses in conjunction with a task requiring orthographic responses (e.g., Perret and Laganaro, 2012), is needed to explore the precise time course of phonological activation in written word production.

As outlined in the Discussion of the first experiment, an alternative explanation of the facilitatory effect in Experiment 1 exists which does not involve phonologically-based priming, but rather exclusively focuses on semantic overlap between the two dimensions. According to this scenario, visual processing of a homophonic distractor results in the co-activation of multiple senses, and in the case of congruent distractors, the co-activated color sense and the conceptual code of the color naming response match. If it is assumed that Stroop effects can arise at a purely conceptual level, then one could account for our facilitatory effects from congruent distractors, without the involvement of phonological codes.

Whether this idea is feasible is not clear. Traditionally, it was postulated that responding in this task was constrained by a response-based bottleneck, i.e., a response buffer with a capacity of only a single word (e.g., Morton, 1969; Posner and Snyder, 1975). Because reading is well known to operate faster than color naming (Cattell, 1886), the word will occupy the response buffer before the color response can be generated. Hence, Stroop interference (word interferes with color naming) arises because the response buffer must be cleared before target color naming can proceed. By contrast, word reading can proceed without interference from the color dimension because the word is produced before the color name can occupy the response buffer (note that the notion of a limited-capacity “response buffer” has recently been re-introduced to explain semantic interference effects in PWI tasks; e.g., Mahon et al., 2007). Subsequent models have tended to focus on attentional control, i.e., word naming is more automatic whereas color naming requires attention. For instance, in Cohen et al.'s (1990) extremely influential article reporting a computational simulation of various Stroop phenomena, two processing pathways, one pertaining to the color and one to the word, cascade activation toward a common response selection layer. In perhaps the most detailed computational analysis of the Stroop phenomenon, Roelofs (2003) extended the WEAVER architecture (Levelt et al., 1999) of spoken word production to the Stroop domain. In this model, verbal response generation involves transmission of activation from conceptual to lexical-syntactic (“lemma”) representations, followed by phonological encoding (access to phonological entries, information about stress pattern, sequence of phonemes, etc.). Critically, the same mechanism which is postulated to underlie general word production (lexical selection via competition) also generates the Stroop effect. Critically, language-based competition is postulated to account even for Stroop results involving manual, rather than verbal, responses (e.g., Sugg and McDonald, 1994), by including an additional layer of response nodes which is outside the language system proper, yet responses are still mediated by the lexical network. Overall, current theoretical and computational models of the Stroop effect tend to locate its origins not at the semantic level, but instead postulate that in some form, a conflict involving response selection is the critical element.

Nevertheless, in order to empirically assess the possibility that the facilitation effect of congruent homophones in our first experiment might have arisen at a conceptual locus, rather than during preparation of the written response, we conducted a second experiment very similar to the first one, but in which the written responses from the first experiment were replaced with a manual classification task. The reasoning here is that effects which have their origin in the language system should be eliminated in a task which does not require verbal responding. In our second experiment, we applied this idea to the Stroop task and asked participants to classify target colors via a key press. If the facilitatory effects of congruent distractors in Experiment 1 are purely conceptually based, then they should still be present in Experiment 2. Contrary to this prediction, we found that the facilitatory effects of (same-tone) congruent homophones observed in Experiment 1 was no longer present in Experiment 2. This finding supports the argument in the previous section that a “purely” conceptual locus of Stroop effects is probably not tenable. By contrast, Stroop interference effect was found which was similar in size to the one in the first experiment. Note that Stroop interference effects showed a similar sensitivity to tone and SOA as in the first experiment: at SOA = 0 ms, interference was much more pronounced for same-tone than for different-tone incongruent characters, but at the negative SOAs, tone appeared less relevant.

In summary, in a Stroop task which involved handwritten responses and Chinese distractors which were congruent or incongruent homophones of color terms, we obtained evidence for a contribution of phonological codes to the preparation of written codes. Future research should aim to further elucidate the specific nature of phonology-to-orthography mappings in written Chinese, the time course of activation of phonological vs. orthographic codes in the preparation of written responses, and the integration of tonal information with phonological codes in Mandarin.

### Conflict of interest statement

The authors declare that the research was conducted in the absence of any commercial or financial relationships that could be construed as a potential conflict of interest.

## Figures and Tables

**Table A1:**
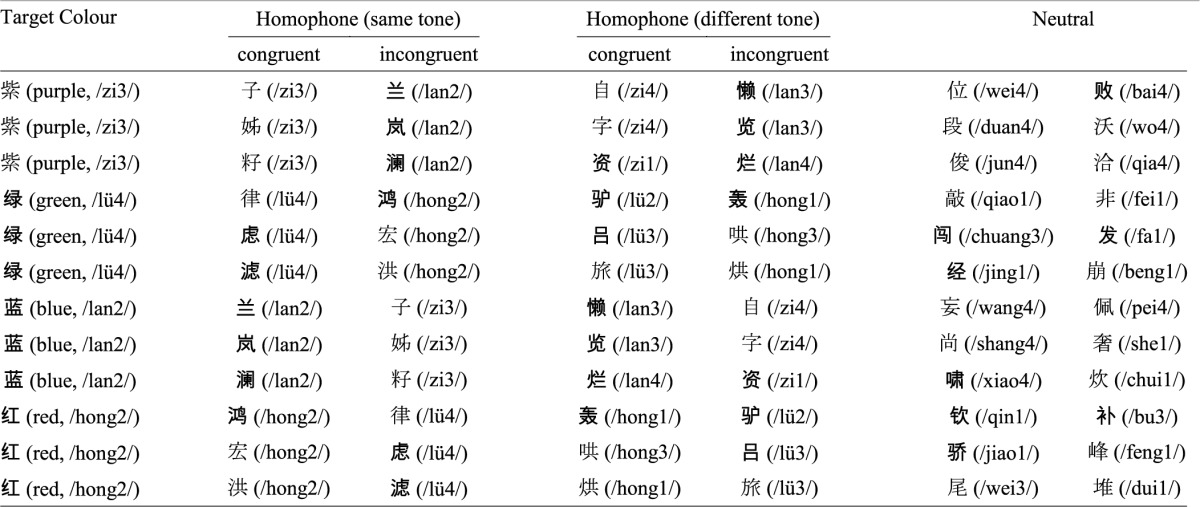
**Stimuli used in Experiment 1 and 2. Given that single Chinese characters are ambiguous in meaning without a dominant meaning in some cases, the translations of characters are not provided**.

## Footnotes

^1^Here and in the results reported thereafter, *N* indicates the number of observations which was included in the model.
